# Nesfatin-1 expression and blood plasma concentration in female dogs suffering from cystic endometrial hyperplasia and pyometra and its possible interaction with phoenixin-14

**DOI:** 10.1186/s12917-024-04336-w

**Published:** 2024-10-25

**Authors:** Marta Rybska, Marek Skrzypski, Maria Billert, Tatiana Wojciechowicz, Anna Łukomska, Piotr Pawlak, Tomasz Nowak, Karolina Pusiak, Barbara Wąsowska

**Affiliations:** 1 Department of Preclinical Sciences and Infectious Diseases, Poznań University of Life Sciences, Wołyńska 35, Poznań, 60-637 Poland; 2 Department of Animal Physiology, Biochemistry and Biostructure, Poznań University of Life Sciences, Wołyńska 35, Poznań, 60-637 Poland; 3 Department of Genetics and Animal Breeding, Poznań University of Life Sciences, Wołyńska 33, Poznań, 60-637 Poland; 4 Department of Physiology and Toxicology, Institute of Animal Reproduction and Food Research of Polish Academy of Sciences, Tuwima 10, Olsztyn, 10-747 Poland; 5 Department of Internal Diseases and Diagnostics, Poznań University of Life Sciences, Wołyńska 35, 60-637 Poznań, Poland

**Keywords:** Nesfatin-1, Phoenixin, Uterus, Reproductive diseases, Canine, BCS

## Abstract

**Background:**

Nesfatin-1 is a neuropeptide that regulates the hypothalamic-pituitary-gonadal axis and may play a role in uterus function. It is co-expressed with other peptides, such as phoenixin, which can influence sex hormone secretion. Our previous research has confirmed that phoenixin-14 is involved in the development of cystic endometrial hyperplasia (CEH) and pyometra in dogs. Therefore, based on the similarities and interactions between these neuropeptides, we hypothesized that nesfatin-1 might also regulate the reproductive system in dogs. This study aimed to determine the expression of nesfatin-1 and its interaction with phoenixin-14 in dogs with CEH or pyometra compared to healthy females, and concerning animals’ body condition score (BCS 4–5/9 vs. BCS > 5/9).

**Results:**

The analysis of nesfatin-1 in the uterus of bitches consisted of qPCR, western blot and immunofluorescence assays, and ELISAs. The results showed significantly higher nesfatin-1 encoding gene, nucleobindin-2 mRNA (*Nucb2*) and nesfatin-1 protein expression in overweight females and those suffering from CEH or pyometra compared to healthy animals. The immunoreactivity of nesfatin-1 was elevated in the uteri of bitches with higher BCS > 5/9. Moreover, nesfatin-1 blood concentrations increased in all examined overweight bitches. In the case of phoenixin signals, we found opposite results, regardless of the female body condition score.

**Conclusion:**

The etiology of CEH and pyometra are not fully known, although we have expanded the level of knowledge with respect to the possible interaction of nesfatin-1 and phoenixin in female dogs’ uteri. They interact oppositely. With increasing female body weight, the expression of nesfatin-1 in the uterus and its peripheral blood concentration increased. However, for female dogs affected by CEH and pyometra, a decreased level of phoenixin-14, irrespective of their body condition score is characteristic. This knowledge could be crucial in the development of biomarkers for these conditions, which may lead to earlier recognition.

**Supplementary Information:**

The online version contains supplementary material available at 10.1186/s12917-024-04336-w.

## Background

Pyometra is a common reproductive disease, occurring mostly during the diestrus phase in elderly bitches. Another prevalent reproductive disorder in dogs, which often corresponds with pyometra but has a non-bacterial origin, is cystic endometrial hyperplasia [1–3]. Despite extensive studies on these diseases, our understanding of their etiology and pathogenesis is still limited [2, 4]. Recently, we found that neuropeptide phoenixin is expressed in the reproductive tract of female dogs. Expression of phoenixin and its putative G protein-coupled receptor 173 (GPR173) are present in the ovary, uterus, and periovarian adipose tissue in female dogs [4]. Furthermore, we found that PNX and GPR173 productions decreased in dogs suffering from CEH and pyometra [5]. Following our previous analyses in the bitch, the presented study aims to explore the potential role of nesfatin-1 and phoenixin-14 in regulating the uterus function in female dogs.

Nesfatin-1 is a neuropeptide composed of 82-amino acids produced from the precursor 396-amino acid protein named nucleobindin-2 (NUCB2) [6]. Though the receptor for nesfatin-1 has not been fully defined, certain data suggest its possible intracellular pathways. Nesfatin-1 acts as a ligand of the metabotropic receptor, which is coupled to a Gi/o protein and/or activated Gs-protein-coupled receptor [7]. When the receptor is activated, it causes the opening of L- and P/Q-type calcium channels, leading to a Ca^2+^ influx in the neuroplasm [6, 8].

Studies have also indicated that nesfatin-1 is produced in peripheral tissues including adipose tissue [9], endocrine pancreas [10] or gastrointestinal tract [11]. A high level of Ncub2 expression was shown in the pancreas and stomach of canine digestive tissue. The evidence suggests that this neuropeptide regulates the canine feeding center and energy balance [12, 13]. Intracerebroventricular (i.c.v.) injection of nesfatin-1 reduces food intake in a dose-dependent manner, while injection of a nesfatin-1-neutralizing antibody increases appetite in rats [6] and simultaneously may decreased thermogenesis in rats [14]. In addition, nesfatin-1 affects glucose homeostasis by stimulating insulin release, with its impact on glucose metabolism partly dependent on the ghrelin receptor [15]. Interestingly, this neuropeptide inhibits the contraction of the gastric smooth muscle in normal and obese rats, which points to its potential role in regulating stomach motility [16].

Growing evidence demonstrates that in addition to body weight and metabolism regulation, nesfatin-1 may be involved in the reproduction systems. Nesfatin-1 is widely expressed in male and female reproduction systems [17]. *Nucb2* mRNA has been detected in the testes of rats, mice, dogs, and humans [18]. The protein NUCB2/nesfatin-1 is found near the seminiferous tubules [19, 20] and in the theca and interstitial cells of the ovary [21]. Nesfatin-1 also plays a role in gonadal development during pubertal transition; its levels increase in rats and premature non-obese girls [22]. Nesfatin-1 has been implicated in controlling the hypothalamic-pituitary axis, gonadal functions or pregnancy [23–26].

Notably, Stein et al. determined that nesfatin-1 may interact with another reproductive peptide, phoenixin [27]. Phoenixin peptide is produced from its precursor protein Smim20 [28] and has two main forms consisting of 14 or 20 amino-acid sequences respectively [29]. Phoenixin and nesfatin-1 are similarly expressed in the hypothalamic–pituitary–gonadal (HPG) axis [30]. Localizations of nesfatin-1 and phoenixin have similar characteristics and are reported in various hypothalamus structures such as the arcuate nucleus (ARC), ventromedial hypothalamus (VMH), paraventricular nucleus (PVN), and lateral hypothalamus (LH) where they show co-expression rates of 86%, 76%, 70% and 70% respectively [31]. This co-expression suggests an interaction between these two peptides, which is further supported by their counterbalancing effects in several physiological processes and also reproductive dysfunctions [31, 32].

Our previous studies showed that phoenixin may contribute both to the physiology and the pathogenesis of the reproductive system in female dogs [4, 5]. By contrast, the role of nesfatin-1 in these processes remains unknown. Therefore, the aim of the study is to evaluate the expression and localization of nesfatin-1 in canine uterine tissue during diestrus, CEH and pyometra as well as its potential interactions with phoenixin-14, in relation to animal’s body condition score (BCS).

## Results

### Expression of Nucb2 in the canine endometrium

**Fig. 1:**
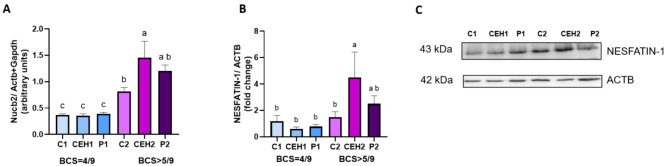
Relative expression of *Nucb2* (**A**) and nesfatin-1 protein production level (**B**) in canine endometrium. The data were normalized to β-actin (*Actb*) and glyceraldehyde-3-phosphate dehydrogenase (*Gapdh*). All treatments were performed in duplicate, and bars represent the mean ± SEM. Representative blots (**C**) for nesfatin-1 (43 kDa) along with ACTB (42 kDa). Different lowercase letters (ab, ac, bc) denote significant differences between each type of group (*p* < 0.05). The same lowercase letters (aa, bb, cc) indicate no significant differences among the study groups (*p* > 0.05). C1/C2: control group; CEH1/CEH2: animals with CEH; P1/P2: animals with pyometra; animals with BCS = 4–5/9: C1, CEH1, P1 groups; animals with BCS > 5/9: C2, CEH2, P2 groups

A higher expression of *Nucb2* was exposed in the endometrium collected from bitches with overweight (C2, CEH2, P2 groups), compared to female dogs with BCS = 4–5/9 (C1, CEH1, P1 groups) (*p* < 0.05). Notably, among overweight bitches, CEH2 group showed the highest *Nucb2* expression in the endometrium compared to the diestrus female dogs (C2 group) (*p* < 0.05) (Fig. 1A). There were no significant differences in *Nucb2* expression levels in endometrium tissue among groups with BCS = 4–5/9 (C1, CEH1, and P1 groups) (*p* > 0.05) (Fig. 1A).

### Protein production of NUCB2/ nesfatin-1 in the canine endometrium

In the endometrium of overweight female dogs with CEH (CEH2 group), there was a higher production of nesfatin-1 protein compared to healthy diestrus animals (C1 and C2 groups, *p* < 0.05) and bitches from CEH1group and P1 group (*p* < 0.05) as shown in Fig. 1B.

Moreover, there were no differences when comparing NUCB2/ nesfatin-1 protein production in endometrium tissue among all groups with BCS = 4–5/9 (C1, CEH1, P1 groups) (*p* > 0.05) (Fig. 1B). As shown in Fig. 1C, representative signals were detected by anti-nesfatin-1 (43 kDa), and anti-ACTB (42 kDa) antibodies.

### Immunolocalization and immunoreactivity of nesfatin-1and PNX-14 in the canine uteri

**Fig. 2:**
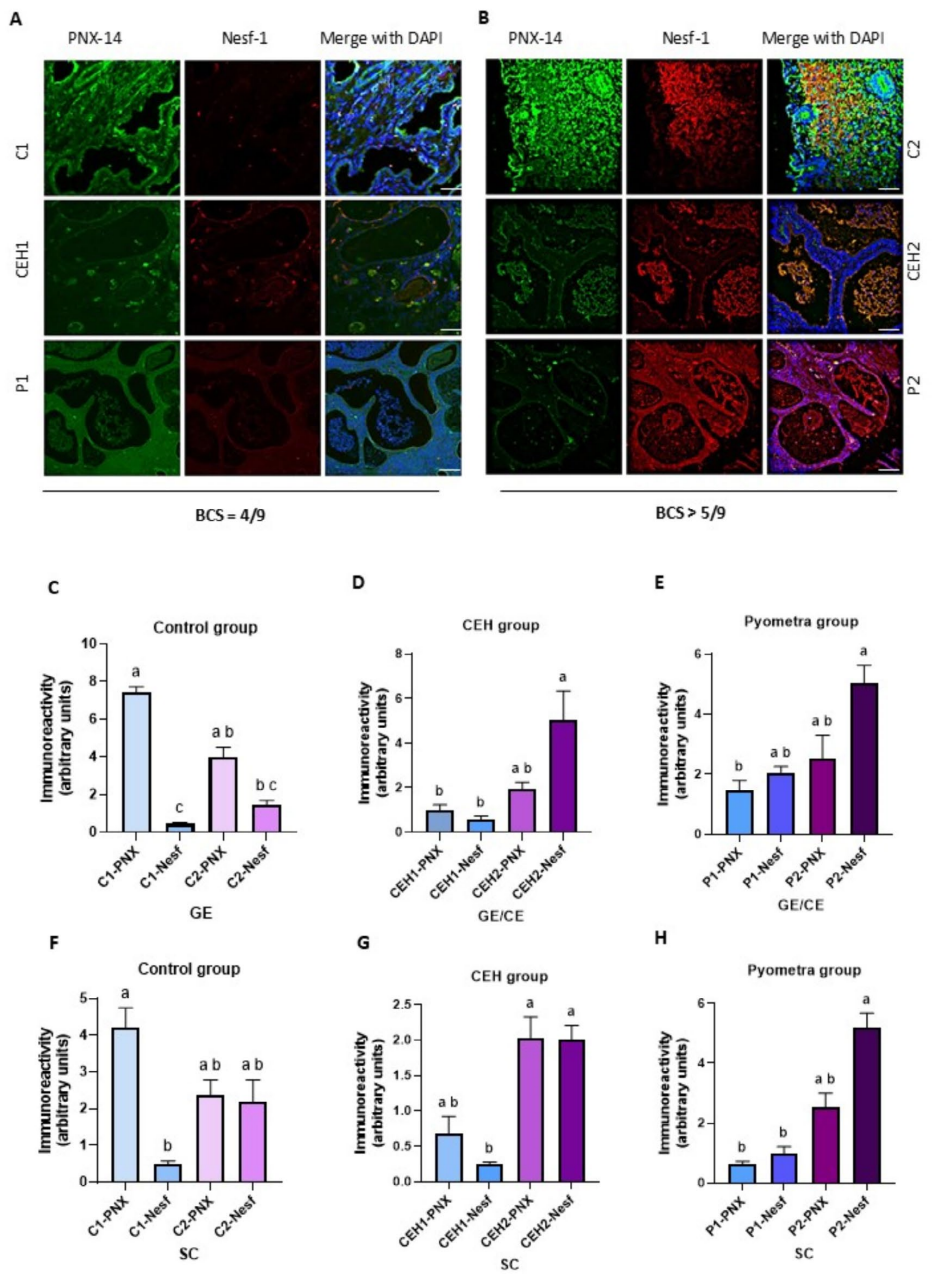
Immunolocalization of nesfatin-1 and PNX-14 proteins in the uterus of bitches with optimal body condition (**A**) and with overweight (**B**). The representative sections of the uterus are presented with PNX-14 (green) and nesfatin-1 (red) immunoreactivity, and nuclei were stained with DAPI (blue). The immunoreactivity levels are presented in the glandular epithelium (GE) (**C**) or glandular epithelium/uterine cyst (CE) (**D**, **E**) and endometrial stroma (SC) (**F**, **G**, **H**). Different lowercase letters (ab, ac, bc) denote significant differences between each type of group (*p* < 0.05). The same lowercase letters (aa, bb, cc) indicate no significant differences among the study groups (*p* > 0.05). Scale bars: 100 μm. C1/C2: control group; CEH1/CEH2: animals with CEH; P1/P2: animals with pyometra; animals with BCS = 4–5/9: C1, CEH1, P1 groups; animals with BCS > 5/9: C2, CEH2, P2 groups

The results indicate that nesfatin-1 signals were most detected in the uteri of animals with confirmed overweight (BCS > 5/9, Fig. 2B) in the control (C2) group and pathological groups (CEH2 and P2) compared to the animals with BCS = 4–5/9 (C1, CEH1 and P1 group, Fig. 2A). The stronger nesfatin-1 signal was identified in the glandular epithelium (GE) and endometrial stroma cells (SC) in the uterus of control animals (C2 group), as well as in the uterine gland secretion and in the wall of endometrial cysts (CE), including within the purulent area (CEH2 and P2 group) (Fig. 2B).

Comparing the immunoreactivity signal of both neuropeptides in healthy endometrium, PNX-14 immunoreactivity was higher in endometrial glands (GE) and endometrial stroma (SC), mainly in animals with BCS = 4–5/9 (C1 group), opposite to nesfatin-1 results, with lower signal density in that group (*p* < 0.05) (Fig. 2C and F).

In animals from CEH2 group, the immunoreactivity of nesfatin-1 in endometrial glands (GE), uterine cysts (CE) and endometrial stroma (SC) significantly increased (*p* < 0.05) compared to the CEH1 group (Fig. 2D and G). Moreover, the immunoreactivity of nesfatin-1 in the endometrial stroma in the dogs affected by pyometra and with overweight (P2 group) was increased compared to those animals with BCS = 4–5/9 (P1 group) (*p* < 0.05) (Fig. 2H). Comparing the signals of both neuropeptides in the pathologically changed uterus, a significantly higher level of nesfatin-1 was observed from the GE and CE structures in animals with overweight (CEH2, P2 groups) compared to lower PNX-14 immunoreactivity detected from uteri of CEH1 and P1 groups (*p* < 0.05) (Fig. 2D and E).

### Concentrations of nesfatin-1, phoenixin-14, and progesterone in canine peripheral blood

**Fig. 3:**
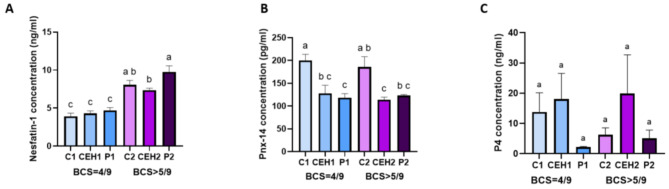
Concentrations of nesfatin-1 (**A**), PNX-14 (**B**) and P4 (**C**) in canine peripheral blood. All analyses were performed in duplicate, and the bars represent the mean ± SEM. Different lowercase letters (ab, ac, bc) denote significant differences between each type of group (*p* < 0.05). The same lowercase letters (aa, bb, cc) indicate no significant differences among the study groups (*p* > 0.05). C1/C2: control group; CEH1/CEH2: animals with CEH; P1/P2: animals with pyometra; animals with BCS = 4–5/9: C1, CEH1, P1 groups; animals with BCS > 5/9: C2, CEH2, P2 groups

Plasma NUCB2/nesfatin-1 concentrations of the healthy female dogs and those suffering from CEH and pyometra are shown in Fig. 3A. Nesfatin-1 concentration was increased in the blood of diestrus dogs (C2 group) or affected by uterus disorders (CEH2 and P2 groups) and with confirmed overweight, compared to animals with BCS = 4/9 (C1, CEH1, P1 groups) (*p* < 0.05).

Our results indicated that regardless of body condition score, PNX-14 concentration in the diestrus female dogs (C1 and C2 groups) was higher compared to the other exanimated groups with uterine disorders (CEH 2, and P1, P2 groups) (*p* < 0.05) (Fig. 3B). Additionally, P4 blood plasma level was determined to range from 2.5 ng/mL to 70 ng/mL. The progesterone and PNX-14 values of healthy diestrus bitches and animals with CEH and pyometra are presented in Fig. 3C. The results of P4 concentration did not show any significant differences among the investigated study group and all female dogs were in the diestrus phase (*p* > 0.05).

Pearson’s correlation coefficients were used to detect changes in the concentration of nesfatin-1 and PNX-14 as well as between nesfatin-1 and P4, BCS, or animal age in the peripheral blood among all studied groups. The obtained results are presented in Table 1. The nesfatin-1 concentration was positively correlated with BCS results (*r* = 0.5757, *p* < 0.001). In the case of other compared parameters, between nesfatin-1 and the investigated group the correlation was observed to trend positive; even though these data were not statistically significant, with *p* > 0.05.

**Table 1:** Correlation analysis among nesfatin-1, PNX-14, BCS and age

	Nesfatin-1 vs. PNX-14	Nesfatin-1 vs. P4	Nesfatin-1 vs. BCS	Nesfatin-1 vs. age
(Pearson r)	0.2302	0.08783	0.5757	0.2088
P value	0.2211	0.6444	0.0009**	0.377
Number of Pairs	30	30	30	30

Table 1. Coefficient of correlation (r) obtained between the nesfatin-1 (ng/mL), PNX-14 (pg/mL) and P4 (ng/mL) concentrations in blood plasma and animal age. The results were calculated with Pearson correlation and analyzed with a t-test. The correlation was statistically significant at *p* < 0.05.

## Discussion

Neuropeptides nesfatin-1 and phoenixin affect the hypothalamic-pituitary-gonadal (HPG) axis and play a significant role in the hormonal regulation of reproductive functions. Therefore, both are localized together in several hypothalamic nuclei and potentially interact [19]. Though there are many studies, there is still a lack of data regarding the expression and functions of those neuropeptides in a canine uterus, particularly in terms of proliferative and degenerative changes. For the first time, our study has shown the expression and localization of nesfatin-1 in overweight canines and those suffering from CEH and/or pyometra. In addition, we tried to find a possible connection between PNX-14 and nesfatin-1 in the development of uterus pathology.

In the healthy dogs or the ones with CEH or pyometra with optimal body conditions, no difference was found in the expression of mRNA or nesfatin-1 protein production. Higher expression of *Nucb2* and its protein was detected only in individuals with overweight and developing pathology disorders in the uterus. These findings strongly support the idea that nesfatin-1 is also a key factor regulating weight and metabolism dysfunction in the dog. As for phoenixin, our previous study showed a decreased level of that neuropeptide in the uteri of female dogs suffering from CEH and/or pyometra. Moreover, lower phoenixin-14 expression and its plasma concentration were detected in female dogs with uterus disorders, regardless of body weight [5]. Our data on neuropeptide in the dog indicated that during the development of uterus pathology conditions, expression of nesfatin-1 increased while the PNX-14 production was down-regulated.

Although, phoenixin and nesfatin-1 are similarly expressed in the hypothalamus area, they may have opposing effects on various physiological processes. Nesfatin-1 has an anorexigenic effect, however the potential nesfatin signaling pathways are not fully recognized [7, 19]. On the other hand, phoenixin has a strong orexigenic effect, meaning that it stimulates food intake. Interestingly, the stimulation of food intake by phoenixin is accompanied by an increase in nesfatin-1 immunoreactivity, suggesting that these two peptides interact in a balancing manner [19]. The hypothalamic centers, where the nesfatin-1 is expressed, such as ARC, PVN, and LH, are in charge of maintaining the body’s energy and osmotic balance and play a role in regulating reproductive processes [18].

In addition to metabolic diseases, NUCB2/nesfatin-1 has been involved in reproductive disorders such as endometriosis and endometrial cancer [33]. Some studies report high expression of NUCB2/nesfatin-1 in type I and type II endometrial cancer tissue [34] while others report an inverse correlation between nesfatin-1 and endometriosis, as serum nesfatin-1 remains low in patients with endometriosis [35]. Similarly, Kulinska et al., [36], found reduced PNX serum concentration and tissue GPR173 expression in women with endometriosis.

The distribution of nesfatin-1 in the uterus was previously determined by immunohistochemical methods and its immunoreactivity was mostly found in the glands and endometrial epithelial cells. Moreover, a higher expression of *Nucb2* mRNA was shown in estrus compared to other phases [21]. To confirm the role of PNX-14 and nesfatin-1 in endometrial changes, the localization of both neuropeptides was observed in rat uterus according to phases of the estrous cycle [30]. The highest level and expression of both neuropeptides in the estrus with reducing concentration was found during the diestrus and then proestrus phase. Nesfatin-1 and PNX-14 immunoreactivity in rats were most detected in glandular and endometrial epithelial cells. However, studies on the possible role of neuropeptides regarding pathological changes of the uterus in other species are still limited.

In this study, we presented for the first time that colocalization of nesfatin-1 and phoenixin in the canine uterus is affected by reproductive system diseases in a body weight- dependent manner. A higher immunoreactivity of nesfatin-1 was found in the uteri of healthy and CEH or pyometra-suffering animals with confirmed overweight. Strong neuropeptide signals were mostly detected from cytoplasm of stroma cells, multifocal cystic glands, and normal glandular epithelial cells. Interestingly, the signal detected in the uteri section from animals with optimal BCS was weak regardless of uteri conditions. These results showed that nesfatin-1 levels increased in the uteri of overweight female dogs, supporting the hypothesis that this neuropeptide may play a role in metabolic regulation and the development of uterine disorders. Additionally, the presented data discovered a higher PNX-14 signal in healthy females compared to bitches with uterine disorders. The immunoreactivity of phoenixin decreases in CEH-affected bitches, regardless of BCS. In pyometra, the uterus may undergo an advanced degenerative and/or inflammatory process that can significantly reduce the expression of PNX in the uterine tissue. These observations were consistent with our previous study, where stronger phoenixin and GPR173 protein signals were detected in both the luminal and glandular epithelial cells of healthy animals and decreased in uteri with pathological changes [5, 6].

Modulation by pro-inflammatory cytokines such as interleukin (IL-6) and tumor necrosis factor-alpha (TNF-α), as well as anti-inflammatory drugs, suggests that nesfatin plays a role in obesity and inflammation [9, 15]. Clinical signs of pyometra may be related to a biological role of nesfatin-1, including inflammatory response, anxiety, lethargy, increased thirst and urination, and moderate to severe depression [37–39]. Moreover, in blood parameters, an increased C-reactive protein (CRP) level is possibly the most prominent biomarker of pyometra [39]. Recent studies confirmed that circulating levels of nesfatin-1 induce fear-potentiated startle responses, depression/anxiety-like behavior, and reduced exploratory behavior in rodents [37, 38]. In mouse adipose tissue, nesfatin-1 expression and secretion increase with diet-induced obesity and are influenced by pro-inflammatory cytokines (IL-6, TNF-α) and insulin. In contrast, food restriction decreases nesfatin-1 mRNA and protein levels [9]. The changing nesfatin level and its peripheral tissue expression may also be correlated with inflammatory markers such as IL-6 and CRP and level of corticosterone in humans [40]. In obese humans with high BMI, there are more nesfatin-1 immunopositive cells and a lower number of ghrelin immunopositive cells in the gastric mucosa [15].

In our study, a higher concentration of nesfatin-1 and its elevated mRNA expression in endometrial tissue was observed in bitches affected by pyometra with clinical signs of this disease and increased BCS. In individuals suffering from pyometra with optimal body conditions, the nesfatin-1 plasma was at a stable level, similar to that of healthy animals. Research has shown that fluctuations in nesfatin levels are a more direct result of metabolic changes than the inflammation process that occurs in the bitches. On the other hand, phoenixin-14 plasma concentration consistently decreased in female dogs suffering from CEH or pyometra, regardless of body condition. However, it may be increased in the presence of ovarian cysts irrespective of the canine uterine condition [5]. Further investigations are required to confirm the possible correlation between nesfatin-1 and BCS, CRP, IL-6, and the presents of ovarian cysts in bitches suffering from pyometra.

The published studies on the correlation between serum levels of nesfatin-1 and BMI are inconsistent. Some research suggests that individuals with normal weight have higher levels of nesfatin-1 in their blood compared to obese individuals. Several studies indicate a negative correlation between nesfatin-1 levels and BMI, body weight, and fat mass in humans with obesity or metabolic syndrome [41]. Certain studies provide evidence of a positive correlation between serum levels of nesfatin-1 and BMI [9, 42, 43]. In our data, a positive correlation between the nesfatin-1 plasma concentration and a dog’s BCS was shown. Our previous study reported a similar relationship between PNX-14 concentration and animals’ BCS [4]. Despite the novelty of this study, several limitations must be considered when interpreting the results. These include a small sample size, variations in the age of the collected individuals, and the inclusion of various breeds. Additionally, the study focused only on female dogs with a body condition score (BCS) of 4–5/9 compared to overweight animals (BCS 6–7/9). Further research is needed to investigate nesfatin-1 levels in dogs with advanced obesity (BCS 8–9/9) or underweight bitches (BCS < 4/9) to fully understand how the physiological status of animals influences the development of reproductive disorders. In the case of obese female dogs, the correlation may be changed. Additionally, when compared nesfatin-1 to phoenixin concentration, P4 level or age, the correlation tends to be positive but not significant statistically.

The findings must be interpreted with caution, considering the limitations discussed above. Nevertheless, this study showed a perspective on the association between nesfatin-1 and CEH or pyometra development and animal’s BCS, marking a significant step forward in understanding the etiology of uterine disorders.

## Conclusion

Nesfatin-1 and phoenixin-14 are two neurotransmitters that may have a role in regulating the reproductive system. Our research revealed the expression and localization of both neuropeptides in canine reproductive organs for the first time. Evidence suggests the opposite involvement of nesfatin-1 and phoenixin-14 in CEH and pyometra development.

Although the exact mechanisms of nesfatin-1 activation are not fully understood, further research is recommended to study the role of nesfatin-1 and PNX-14 in canine reproductive organs in more-detailed fashion, especially in the development of biomarkers for CEH and pyometra conditions.

## Methods

### Animals and samplings

The animals accepted in the study underwent precise clinical assessment before the routine ovariohysterectomy (OVH) procedure, performed at the request of the owners. All collected samples were obtained from 68 female dogs with optimal body conditions and/or with confirmed overweight. All OVH procedures were performed in the veterinary clinic My Pet in Poznań (Poland) and the University Centre for Veterinary Medicine in Poznań (Poland). The tissue and blood samples were collected according to standard veterinary protocols with written owner’s consent. The surgical procedures were performed under general anesthesia. Prior to surgery, all dogs were administered pre-medication of 5 µg/kg iv dexmedetomidine (Dexdomitor^®^ Zoetis, Warsaw, Poland) and 0.3 mg/kg iv methadone (Comfortan^®^, Dechra, Warsaw, Poland). Each animal was induced with an administration of 3 mg/kg of iv propofol (Propomitor^®^ Orion Pharma Warsaw, Poland) and then the anesthesia was maintained by inhalation of isoflurane in oxygen (Vetlurane^®^, Virbac, Warsaw, Poland). To manage postoperative pain before the surgery, subcutaneous injections of meloxicam (0.2 mg/kg, Metacam^®^ Boehringer Ingelheim, Warsaw, Poland) were administered. On the day of surgery, all female dogs received intravenous continuous rate infusion (CRI) of multi-electrolyte fluid (Optilyte^®^, Fresenius Kabi, Warsaw, Poland). During the first three days post-surgery, all female dogs received p.o. 0.1 mg/kg of meloxicam (Meloxidy^®^, Ceva, Warsaw, Poland). Subcutaneous administration of 12.5 mg/kg of amoxicillin with clavulanic acid (Synulox^®^, Zoetis, Warsaw, Poland) was given prior to OVH and then continued at the same dose twice daily orally for five days.

Specific details about the examined female dogs are presented in Table S1. All bitches were directly examined by ultrasonography to evaluate uterine lesions. Firstly, due to their body condition, the female dogs were divided into two main groups, with optimal body weight (BCS range 4–5/9) and with confirmed overweight and BCS range 6–8/9. Due to the very limited number of female dogs with confirmed uterine disorders and BCS lower than 4/9 or BCS > 8/9, those animals were excluded from the study. Progesterone concentration was also analyzed, and only diestrus dogs with P4 concentration > 2ng/mL and confirmed by vaginal cytology diestrus phase, were included in the research. Animals with other pathological conditions, apart from CEH or pyometra, as well as those previously treated with hormone therapy, were excluded from the study. After the OVH procedure, macroscopic characteristics of the uterine horns, that were used to classify the individual group, such as the degree of redness and edema of the endometrium, the presence of cysts on the endometrial surface, and the presence and color of discharge were identified.

### The animals were divided into six groups

- C1 (*n* = 12), control 1 group, consisting of healthy diestrus dogs with optimal body conditions, BCS = 4–5/9.
- C2 (*n* = 12), control 2 group, a group of healthy animals in the diestrus phase with overweight and BCS > 5/9 (BCS range 6–8/9).
- CEH1 (*n* = 12), represents bitches in the diestrus phase with confirmed CEH (cystic endometrial hyperplasia) and with optimal body conditions, BCS = 4–5/9 with no significant abnormalities in blood parameters.
- CEH2 (*n* = 12), represents bitches in the diestrus phase with confirmed CEH and with overweight, BCS > 5/9 (BCS range 6–8/9). The histological characteristics of collected uteri issues were the same as in the CEH1 group.
- P1 (*n* = 12), pyometra group1, consisting of dogs with pyometra confirmed by ultrasound examination. Seven females were classified as closed-cervix pyometra, and five were qualified as open-cervix pyometra and had a purulent vulvar discharge. All animals were in the diestrus phase, with optimal body conditions with BCS = 4–5/9. Uterine ultrasonography showed the presence of irregular and hypertrophic endometrium and fluid-filled colored discharge uterus.
- P2 (*n* = 8), pyometra group 2, collected from overweight dogs with pyometra and assessment of BCS > 5/9 (BCS range 6–8/9). Five females were classified as having closed-cervix pyometra, and three as having open-cervix pyometra. Clinical diagnosis of canine pyometra, uterine ultrasonography, and histopathological characteristics was the same as in group P1.

Following OVH surgery, the uterus and ovaries were transported to the laboratory. Next, the middle fragment of the uterine horn was removed, dissected, and stored at -80 ^o^C for further gene expression analysis. Additionally, uterine tissue fragments were collected for histopathological and immunofluorescence analysis. Before OVH procedure, blood samples were collected from saphenous veins into K2/K3_EDTA tubes (Sarstedt INC., Germany). The plasma samples were obtained by centrifuging EDTA tubes at 500× g for 15 min at 4 °C and were stored at -20 °C until assayed.

### Histological examination of uteri

After macroscopic examination, the qualification to selected groups was verified by histological analysis of uterine slices, performed by a veterinary pathologist according to the De Bosschere and Wozna-Wysocka classification [44, 45]. Fragments of the middle sections of the uterine horns (approximately 2–3 cm) were fixed in 10% buffered formalin. The sections were stained with H&E using histological procedures, as we previously describe [5]. Tissue slice evaluation was performed using an Axio Lab. A1 microscope (Carl Zeiss Microscopy, Jena, Germany). The presented pictures were analyzed using computer software (ZEN 3.8 (blue edition), Carl Zeiss Microscopy, 2011).

### Characteristics of histological examination of collected tissues

In control groups (C1, C2), histological analysis of uterine slices did not confirm any inflammatory alterations, and the image of the endometrium was characteristic of the middle – and late-diestrus phases of the estrous cycle.

In animals with cystic endometrial hyperplasia (CEH1 and CEH2 groups), the histological examination confirmed the changes in endometrium structure, such as endometrial edema, the proliferation of endometrial glands, and the formation of numerous multifocal serum-filled cysts. Additionally, major progressive atrophy of glandular epithelium was observed. There was no infiltration of inflammatory cells in microscopic analyses.

In pyometra-suffering animals (P1 and P2 groups), chronic, purulent endometriosis has been observed. During the histological analyses of uterine slices, the presence of inflammatory cells (neutrophils, lymphocytes) in the endometrium was identified. Moreover, multifocal endometrial gland hyperplasia in most individuals and a few cases of mild endometrial fibrosis and endometrial hemorrhage were found. Representative photomicrographs of H&E staining of canine endometrial tissue and results of histological examination are presented in Supplementary file (Fig. S1).

### Gene expression analyses

#### RNA isolation and cDNA synthesis

The RNA was obtained from 50 mg of canine endometrium and was homogenized with 1 mL of TRI-Reagent solution (Sigma, T9424, Darmstadt, Germany) by a Tissue Lyser LT bead homogenizer (Qiagen, Germany). Total RNA was extracted according to the manufacturer’s instructions (Sigma, Darmstadt, Germany), and the quality and quantity of RNA were measured spectrophotometrically (NanoPhotometer^®^NP-80; IMPLEN, München, Germany). Only samples having an A260/A280 ratio of between 1.8 and 2.0 were accepted for further analysis. In the next step, the sample of RNA (1 µg) was reverse-transcribed to cDNA using the Transcriptor First-Strand cDNA Synthesis Kit (Roche Diagnostics, Indianapolis, USA). The reaction was processed with the supplier’s protocol and involved the following steps: 65 °C for 10 min, 55 °C for 30 min, 85 °C for 5 min, and stopped at 4 °C. The obtained cDNA was stored at − 20 °C for RT-qPCR analysis.

### RT-qPCR

To analyze the expression level of the *Nucb2* gene in the canine uterus, a real-time RT-PCR assay was performed. The sequences of selected primers for *Nucb2* (nesfatin-1 encoding gene) and the housekeeping gene: glyceraldehyde 3-phosphate dehydrogenase *(Gapdh*) and β-actin (*Actb*) used as an endogenous control are presented in Table S2 (Supplementary File). The RT-qPCR reactions were performed in duplicate on the CFX OPUS 384 (Bio-Rad, California, USA). The reactions (with 20 µL volumes) were conducted using 10 µL EvaGreen qPCR Mix (Solis BioDyne, Tartu, Estonia), 1 µL cDNA, 1 µL of each primer (5 pmol), and 7 µL distilled water. The reaction condition was as follows: 45 cycles of denaturation-10s at 95 °C, annealing- 30s at 60 °C, elongation- 1s at 72 °C and a final extension step for 30s at 60 °C followed by a melting curve analysis to confirm the specificity of amplified products. A negative control reaction, consisting of Rnase-free water instead of cDNA, was used in each measurement. The relative quantification of gene expression levels was assessed using ΔΔCt methods.

### Western blotting

Protein isolation and western blot procedures were performed as previously described [5]. The collected endometrium samples (20 mg) were homogenized in 500 µl of RIPA buffer (R0278, Sigma, Darmstadt, Germany) containing protease and phosphatase inhibitor cocktails (Roche Diagnostics, Indianapolis, USA). The membranes were incubated (overnight at 4 ^o^C) with anti-nesfatin-1 (1:500 dilution, PA5-41311, Invitrogen, Rockford, Illinois, USA) antibody in TBST supplemented with 1% BSA and next with the secondary goat anti-rabbit IgG HRP antibody (1:5000 dilution, sc-2004; Santa Cruz Biotechnology, Dallas, Texas, USA) for 1 h at RT. After stripping the membrane, β-actin immunodetection was performed (1:1000 dilution, sc-47724, Santa Cruz Biotechnology, Dallas, Texas, USA) and used as the loading control. Signals were visualized by chemiluminescence using the Clarity Western ECL Substrate (BioRad, California, USA), and images were captured by the ChemiDoc Imaging System (BioRad Laboratories, Munich, Germany). The intensity of bands was quantified using Image Lab Software (Bio-Rad Laboratories, Munich, Germany).

### Immunofluorescence staining

The immunofluorescence staining of nesfatin-1 and phoenixin-14 procedure was performed as previously described [5, 46]. To expand our previous data-reported PNX-14 localization in the canine uterus [5], we added this neuropeptide to the presented analyses. The selected uterus slices (5 μm) after deparaffinization and rehydration were incubated with a blocking buffer containing 10% normal rabbit serum, and 1% BSA solution in PBS for 2 h at RT. Next, slides were incubated with an anti-nesfatin antibody (PA5-41311, Invitrogen, Rockford, Illinois, USA, ) diluted at 1:250 in PBS and anti-PNX-14 (1:250 dilution, H-079, Phoenix Pharmaceuticals Inc., Burlingame, California, USA) overnight at 4 ^o^C. After the washing procedure in TBST, the slices were incubated with a secondary Alexa Fluor 480 secondary antibody (1:500 dilution) for phoenixin-14 and Alexa Fluor 555 anti-rabbit (diluted 1:500 in TBST) for nesfatin-1, for 1 h at RT in darkness. Afterwards, the tissue slides were air-dried and covered with the histology mounting medium Fluoroshield™ with DAPI (F6057, Sigma Aldrich, Darmstadt, Germany), used for nuclear staining. Tissue sections were analyzed using a confocal microscope Zeiss LSM 880 (Carl Zeiss, Oberkochen, Germany).

Immunoreactivity (optical density) of fluorescence signals was assessed by irradiating canine uterine tissue samples. Analysis of the immunofluorescence signals was conducted using ImageJ/Fiji software (NIH Image, National Institutes of Health, Bethesda, Maryland, USA). At least three distinct areas per slide were analysed. The macro area analysed was a rectangle (100 mm²), covering approximately 25% of the image area (400 mm²). The microscopic analyses included different tissue regions: luminal epithelium (LE), endometrial glands (GE), endometrial cysts (CE) (in pathological uteri only- CEH, pyometra), endometrial stroma (SC). For each group (C1/C2, CEH1/CEH2, P1/P2), the signal from GE/CE cells and SC was analysed and presented as mean ± SEM in arbitrary units.

To validate the nesfatin-1 antibody, we detected signals in the rat hypothalamus as a positive control (Fig. S2a). Additionally, to verify the specificity of the used secondary antibody, a negative control for each slice: a solution of 1% BSA in 0.1 M PBS equivalent to the primary antibody, was performed (Fig. S2b).

### ELISA, RIA

Measurements of P4 in blood plasma were performed using a radioimmunoassay (DIA Source PROG-RIA-CT Kit; DIA source ImmunoAssays S.A., Belgium) according to the manufacturer’s protocol, with a sensitivity of 0.19 ng/mL. All progesterone analyses were measured in duplicate. The intra-assay coefficients of variation (CV) were < 5%.

Nesfatin-1 concentration in blood plasma samples was measured using a commercial Canine Nesfatin-1 ELISA Kit (E0141Ca, BT LAB, China) according to the manufacturer’s protocol and the intra-assay CV was < 8%. Absorbance values were measured by a Synergy 2 Multi-Mode Microplate Reader (BioTek, Winooski, Vermont, USA) at 450 nm.

### Statistical analysis

Statistical analysis was performed using GraphPad Prism (GraphPad PRISM, Version 9.0, San Diego, CA, USA). All data were also tested for the assumptions of normality using the Shapiro-Wilk test. One-way ANOVA followed by Tukey’s post hoc test was used to determine the relative transcript and protein production of nesfatin-1 in canine uterus neuropeptide blood concentration. Immunoreactivity of nesfatin-1 analyses was performed by the Kruskal-Wallis test and Dunn’s posthoc test. Results are expressed as the mean ± SEM and statistical significance was defined as *p* < 0.05.

The correlation coefficient (r) was compared in selected groups and comparison among groups, nesfatin-1/PNX-14 and nesfatin-1/P4 concentrations and nesfatin-1/BCS were analyzed using t-tests and Wilcoxon matched-pairs signed rank tests. For all analyses, a *p* value < 0.05 was considered to be statistically significant.

## Electronic supplementary material

Below is the link to the electronic supplementary material.

Supplementary Material 1

## Data Availability

All data are included in this published article. The raw datasets are available from the corresponding author on reasonable request.

## Abbreviations

CEH: Cystic endometrial hyperplasia
BCS: Body condition scoring
PNX: Phoenixin
GPR173: G protein-coupled receptor 173
NUCB2: Nucleobindin-2
P4: Progesterone
PVN: Paraventricular nucleus
ARC: Arcuate nucleus
VMH: Ventromedial hypothalamus
PVN: Paraventricular nucleus
LH: Lateral hypothalamus
OVH: Ovariohysterectomy
H&E: Hematoxylin and eosin staining
HPG: Hypothalamic-pituitary-gonadal axis
IL-6: Interleukin 6
TNF-α: Tumor necrosis factor typeα
CRP: C-reactive protein
