# Supplementary material for: Nesfatin-1 expression and blood plasma concentration in female dogs suffering from cystic endometrial hyperplasia and pyometra and its possible interaction with phoenixin-14
Source: BMC Vet Res. 2024 Oct 25;20:486. doi: 10.1186/s12917-024-04336-w (PMC11520108; doi:10.1186/s12917-024-04336-w)
Supplement: Supplementary file 1 — Supplementary Material 1 [file 12917_2024_4336_MOESM1_ESM.docx]

**Supplementary files**

| Item | **C 1** | **C 2** | | **CEH1** | | **CEH2** | | **P1** | | **P2** | |
| --- | --- | --- | --- | --- | --- | --- | --- | --- | --- | --- | --- |
| Number of dogs | 12 |  | 12 | | 12 | | 12 | | 12 | | 8 |
| BCS | 4-5/9 |  | >5/9 | | 4-5/9 | | >5/9 | | 4-5/9 | | >5/9 |
| Body weight (kg) |  |  |  | |  | |  | |  | |  |
| mean ± SEM | 12.07±2.73 |  | 18.5±2.5 | | 16.5±3.9 | | 19.5±2.7 | | 16.24±3.17 | | 20.5±3.4 |
| Age (years) |  |  |  | |  | |  | |  | |  |
| mean ± SEM | 2.5±0.30 |  | 3±0,5 | | 6±1.75 | | 7±1.23 | | 7±1.25 | | 10±0.92 |
| Breed |  |  |  | |  | |  | |  | |  |
| mixed-breed | 5 |  | 6 | | 4 | | 2 | | 4 | | 3 |
| in breed type | 7 |  | 6 | | 8 | | 10 | | 8 | | 5 |

**Table S1.** Details of the animals used in experiments

C: control group; CEH: cystic endometrial hyperplasia, P: pyometra;

C2, CEH2, P2: animals with BCS >5/9 (BCS range 6-8/9)

Number of animals with BCS <4/9: all groups, n=0;

Number of animals with BCS =4/9: C1 group, n=10; CEH1 group, n=8; P1 group, n=9

Number of animals with BCS =5/9: C1 group, n=2; CEH1 group, n=4; P1 group, n=3

Number of animals with BCS =6/9: C2 group, n=5; CEH2 group, n=6; P2group, n=3

Number of animals with BCS =7/9: C2 group, n=7; CEH2 group, n=4; P2 group, n=3

Number of animals with BCS =8/9: C2 group, n=0; CEH2 group, n=2; P2 group, n=2

Number of animals with BCS =9/9: all groups, n=0

**Table S2.** Oligonucleotide sequences

| **Gene name** | **Primers sequence** | **Reference** |
| --- | --- | --- |
| *Nucb2* | F:5’- CAAGTGATTGATGTGCTGGAA -3’  R:5’- GCCACTTGTTGTCTTTTCAGTTC -3' | Nozawa et al., 2016  [13] |
| *Gapdh* | F:5'-GGGTCATCATCTCTGCTCCT-3'  R:5'-AGTGGTCATGGATGACTTTGG-3' | Rybska et al., 2022 [5] |
| *Actb* | F:5'-CTGGACTTCGAGCAGGAGAT-3'  R:5'- GATACCGCATGATTCCATCC-3' | Rybska et al., 2022 [5] |

**Fig. S1.** Representative photomicrographs of the canine uterus tissue, H&E staining

**
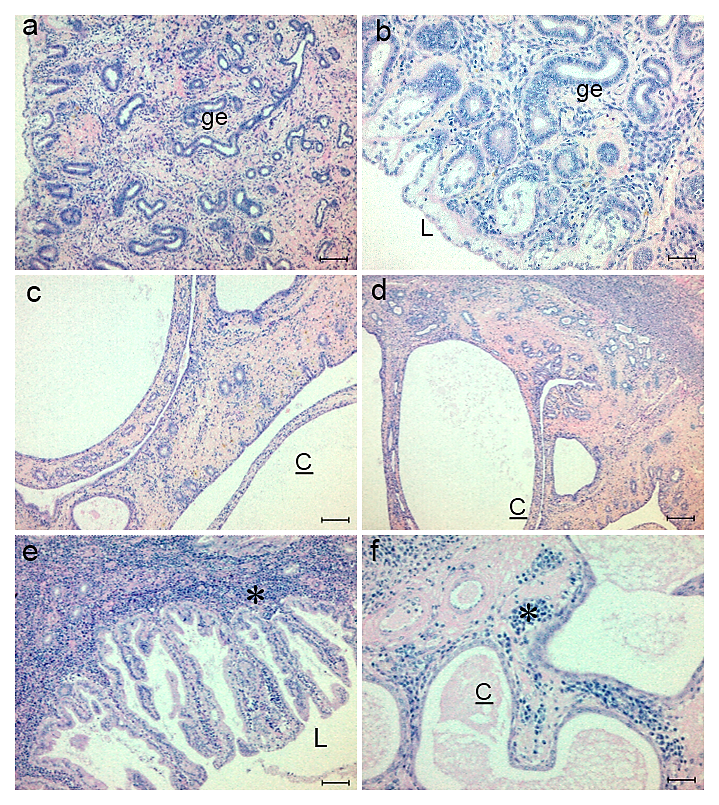
**

Abbreviations: a,b: uterus of control animals in diestrus; c,d: uterus of bitches suffer from CEH, e,f: uterus with pyometra; L: uterus lumen; ge: endometrial glands, C: uterus cysts; asterisk: neutrophils, scale bars: 200 µm

**Fig.S2a.** Representative photomicrographs of nesfatin-1 and phoenixin-14 immunofluorescence signals in rats' hypothalamus


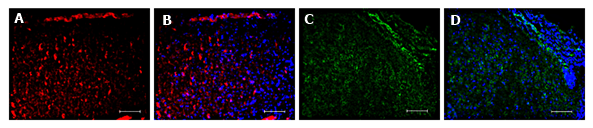


Abbreviations: A: Nesfatin-1, B: Nesfatin-1 with DAPI, C: Phoenixin-14, D: Phoenixin-14 with DAPI. Scale bar:100 µm

**Fig.S2b.** Representative photomicrographs of nesfatin-1 and phoenixin-14 immunofluorescence negative control staining in the uterus section


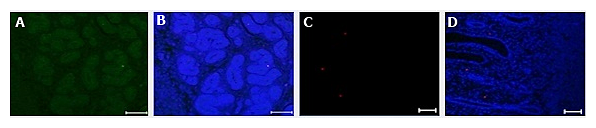


Abbreviations: A: Nesfatin-1, B: Nesfatin-1 with DAPI, C: Phoenixin-14, D: Phoenixin-14 with DAPI. Scale bar:100 µm

**Fig. S3**. Full-length figure for nesfatin-1 (A, 43kDa) and ACTB (B, 42 kDa) protein production.

**A B**

**M 1 2 3 4 5 6 7 8 9 M 1 2 3 4 5 6 7 8 9**


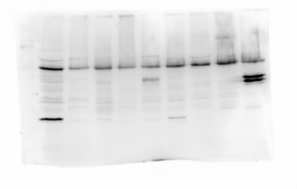

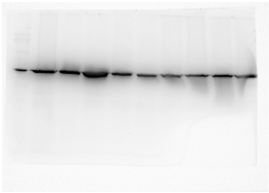


Abbreviations: A: Nesfatin-1image blot, B: β-actin image blot, Frame: represent image blots of Fig.1C; M: protein marker; Line 4: C1, healthy animals, control group, BCS =4/9; Line 5: CEH1, animals with confirmed CEH, BCS =4/9; Line 6: P1, female dogs suffering from pyometra, BCS =4/9; Line 7: C2, healthy animals, control group, and BCS >5/9; Line 8: CEH2, animals with confirmed CEH and BCS>5/9; Line 9: P2, female dogs suffering from pyometra and with BCS> 5/9; Lines 1,2,3: represent as follow P2 group, C1 group and C2 group.
